# A Case of Leukemic Pleural Infiltration in the Blast Crisis Phase of Chronic Myeloid Leukemia: An Unusual Extramedullary Involvement

**DOI:** 10.7759/cureus.60121

**Published:** 2024-05-11

**Authors:** Lakshmipriya V, MonishaRita Jayaraman, Kavitha Kannan, Lakshmi Priya

**Affiliations:** 1 Pathology, Saveetha Medical College and Hospital, Saveetha Institute of Medical and Technical Sciences, Saveetha University, Chennai, IND

**Keywords:** cytology, extramedullary involvement, pleural effusion, blast crisis, chronic myeloid leukemia

## Abstract

The development of pleural effusion in chronic myeloid leukemia (CML) is not well-understood and rarely documented in literature. Extramedullary involvement (EMI), which occurs in about 10% of CML cases, typically affects lymph nodes and the spleen. Instances of extensive infiltration of leukemic cells into the pleura are infrequently reported in CML. Here, we report a case of 41-year-old man experiencing significant bilateral pleural effusion with leukemic infiltration during the blast crisis (BC) phase of refractory CML. Examination of the pleural fluid revealed cells with morphological characteristics of myeloblasts. Although very rare, pleural leukemic infiltration should be considered as a cause of pleural effusion in patients with CML, especially in the BC phase.

## Introduction

Chronic myeloid leukemia (CML) is a condition originating in the bone marrow but can extend beyond it with extramedullary involvement (EMI), infiltrating any organ systems. Typically, this manifests as a tumor known as granulocytic sarcoma or chloroma, although diffuse infiltration without a discernible mass lesion is also possible. The occurrence of EMI in CML is commonly associated with the blast crisis (BC) phase [1]. At the time of CML diagnosis itself, some individuals might experience BC. The occurrence rate of this situation ranges from 0.9% to 6.7% [2]. Most hematological and lymphoid malignancies may sometimes manifest or develop pleural effusions during the clinical progression of the disease. Hodgkin's and non-Hodgkin's lymphomas are most common disorders among them, occurring at a rate of 20-30%, particularly when there is mediastinal involvement [3]. In CML, EMI is observed in 10% of cases, predominantly involving lymph nodes and the spleen. Conversely, occurrences of pleural and peritoneal involvement are infrequent.

Potential causes of exudative pleural effusion in patients with CML encompass a variety of factors, such as leukemic cells infiltrating the pleura, extramedullary hematopoiesis, non-malignant origins, and drugs such as dasatinib, etc. [4]. Dasatinib, an oral inhibitor targeting abl and Src family of kinases (SFK), has gained approval from the US Food and Drug Administration for managing CML patients who have either not responded to or developed resistance against imatinib. Pleural effusion has been noted in 14%-30% of patients, particularly among those receiving dasatinib therapy during BC phase, with severe grade 3-4 effusion observed in 28% of cases in myeloid BC [5]. We present a compelling case of 41 year old man who exhibited a significant bilateral pleural effusion with leukemic infiltration during the BC phase of refractory CML treated with tyrosine kinase inhibitors.

## Case presentation

A 41-year-old man was admitted to our emergency department in the month of March 2023 in intubated state and ionotropic support for further palliative care. He was diagnosed outside with CML in the year 2020 with BCR/ABL gene rearrangement and had recurrent episodes of BC and was treated with multiple failed lines of chemotherapy and tyrosine kinase inhibitors (dasatinib, bosutinib, and imatinib). He also had massive splenomegaly, which was also diagnosed outside and underwent splenectomy in the month of January 2023 for palliation. Histopathological report of the spleen was given outside as extramedullary hematopoeisis with myeloid infiltrate. In view of difficulty in weaning off from ventilator support, tracheostomy was done in our hospital. He also had sepsis with Pseudomonas infection confirmed by blood culture and was started on intravenous antibiotics. Table 1 provides a summary of complete blood count parameters with its reference range.

**Table 1:** Complete blood count findings with the reference range.

Parameters	Value	Reference range
Hemoglobin (g/dL)	6.9	13-17
Red blood cell count (million/cu.mm)	2.57	4.5-5.5
Platelet count (lakhs/cu.mm)	0.23	1.5-4.1
White blood cell count (cells/cu.mm)	132,260	4000-11,000

**Figure 1:**
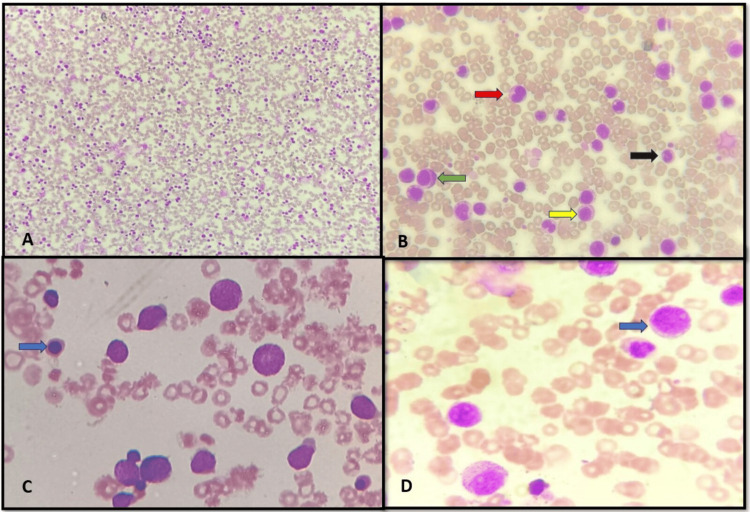
Peripheral smear. (A) x40 increased total white blood cell count, (B) x100 stages of myeloid series: promyelocyte (green arrow) myelocyte (yellow arrow) metamyelocyte (red arrow) and band form (black arrow), (C) x400 nucleated red blood cells (blue arrow), and (D) x400 myeloblast (blue arrow).

Differential white cell count on peripheral smear: neutrophils 10%, lymphocytes 6%, monocytes 1%, eosinophils 2%, basophils 1%, metamyelocytes 14%, myelocytes 11%, promyelocytes 10%, and blast count of 45%. The blood film was consistent with blast crisis of CML, as seen in Figure 1.

**Figure 2:**
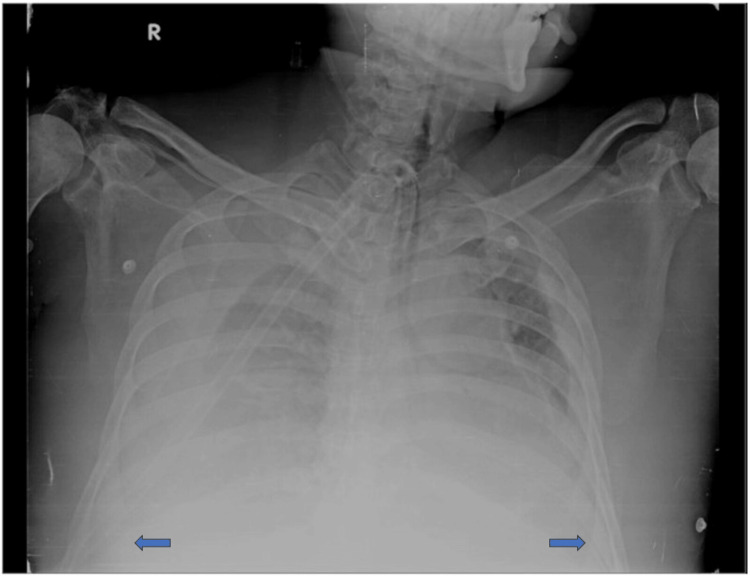
Chest X-ray PA view suggestive of significant bilateral pleural effusion (blue arrow).

Chest X-ray PA view showed significant bilateral pleural effusion, as seen in Figure 2.

**Figure 3:**
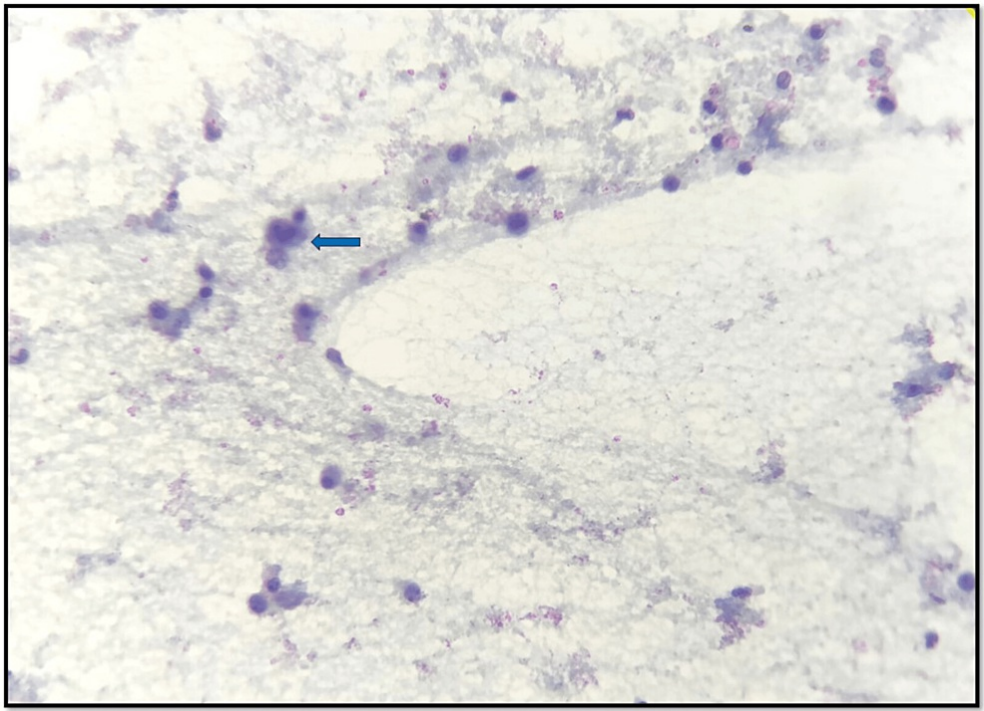
Pleural fluid cytology examination showing lymphocytes and few atypical cells probably blasts (blue arrow).

Based on the imaging result, he was subjected to a diagnostic thoracentesis, which yielded 900 mL of slightly turbid dark red liquid with 2 g/dL total protein, 84 mg/dL glucose, and 6.75 IU/L adenosine deaminase. Pleural fluid cytology showed predominantly lymphocytes, few scattered mesothelial cells, neutrophils, and singly scattered immature myeloid cells, including abnormal large cell, which were morphologically identical to a blast cell, as seen in Figure 3.

The staining for acid-fast bacilli and Gram stain conducted on the pleural fluid yielded negative results. Microbial screening culture of the fluid identified no organisms. The patient was diagnosed with the BC phase of refractory CML complicated with pleural effusion showing leukemic infiltration.

## Discussion

CML is a rare leukemia with an incidence of one per 100,000 population usually diagnosed in the fifth and sixth decade of life with higher incidence in males. The Philadelphia chromosome characterised by t(9;22) translocation is the genetic hallmark of the disease [6]. According to fifth edition of the World Health Organization classification of haematolymphoid tumors, criteria for BC phase of CML include (1) ≥20% myeloid blasts in the blood or bone marrow; 2) the presence of an extramedullary proliferation of blasts; or (3) the presence of increased lymphoblasts in peripheral blood or bone marrow. The optimal cutoff for lymphoblasts and the significance of low-level B-lymphoblasts remain unclear and require additional studies [7]. The extramedullary BC in CML is characterized by the invasion of leukemic blasts into locations outside the bone marrow. This occurrence has been documented in a range of 4%-16% of CML cases over the course of the disease [8]. There are various potential mechanisms, which may underlie exudative pleural effusion in patients with CML. One such mechanism involves infiltration of leukemic cells into the pleura, typically occurring around the time of or before the bone marrow progresses to the BC phase. Leukemic infiltration often impacts areas such as lymph nodes, bones, and the nervous system. While infiltration of organs such as the brain, skin, testes, breast, soft tissues, gastrointestinal tract, synovial membrane, kidneys, ovaries, and pleura is less common, analysis of pleural fluid showed a range of granulocytes at different stages along with a few leukemic blasts [3].

The other mechanism involves a pleural reaction triggered by bleeding into the pleural cavities, leading to pleural effusion in CML patients. Factors such as leukostasis and platelet dysfunction may contribute to the development of hemorrhagic pleural effusion. In that case, the ratio of red blood cells to nucleated cells in the blood and effusion should be similar. In our patient, the ratio of red blood cells to nucleated cells was greater in the blood compared to the pleural effusion. Therefore, it is probable that the abundant nucleated cells present in the pleural fluid originated within the pleural cavities. The third potential mechanism contributing to effusion in patients with CML involves pleural extramedullary hematopoiesis. This condition can present as a distinct mass in various organs such as the liver, spleen, lymph nodes, kidneys, breasts, thyroid, pancreas, endometrium, and mediastinum, or it may be evident in serous effusions. Unlike pleural leukemic infiltration, extramedullary hematopoiesis involves hematopoietic cells from all three lineages, including erythroid, myeloid, and megakaryocytic, although one lineage may be predominant. A study categorizing the cytomorphologic characteristics of extramedullary diseases in 18 patients with CML identified three types based on the ratio of blasts to differentiated granulocytic cells. These categories included blastic, characterized by a predominance of blasts (five patients, 28%); immature, marked by a predominance of blasts and other non-blastic myeloid precursors (eight patients, 54%); and mature, featuring a complete range of granulocytic maturation from blasts to granulocytes (five patients, 28%). Another potential mechanism for effusion involves nonmalignant factors, such as infection. Hence, it is crucial to rule out the possibility of an infectious process. The detection of necrotic debris or the positive identification of microorganisms using specific staining techniques may suggest an infectious cause [3,9]. 

Dasatinib, an inhibitor of the BCR-ABL1 tyrosine kinase, is part of a powerful group of drugs capable of significantly extending the life expectancy of individuals with chronic myelogenous leukemia by inhibiting the fusion protein responsible for cell proliferation. While the precise reasons for the development of effusions in patients treated with dasatinib are not fully understood, there are indications that its impact on the immune system's off-target effects might be involved. This is suggested by occasional instances of large granular lymphocytosis observed in peripheral blood tests of dasatinib-treated patients, marked by the clonal expansion of natural killer and T cells [10]. 

A case report by Hu et al. stated that patient initially presented with pleural effusion, which subsequently led to the diagnosis of CMML-0 stage. Research indicates that approximately 1.7% of CMML patients present with serosal effusion at the time of diagnosis, with a prevalence of one in 60 cases [11]. After reviewing the literature and considering the clinical evidence, the cause of pleural effusion in our patient was attributed to leukemic infiltration into the pleura.

## Conclusions

In summary, patients diagnosed with CML should be monitored for the potential emergence of extramedullary manifestations of BC, particularly in the chronic phase. When encountering a CML patient presenting with pleural effusion, potential causes such as extramedullary hematopoiesis and bleeding into the pleural cavity should be initially investigated. Upon ruling out these causes, the presence of leukemic cells in the effusion might indicate leukemic infiltration of the pleura. Pleural infiltration by CML adversely affects prognosis.
